# Where are the most informative neurons?

**DOI:** 10.1186/1471-2202-14-S1-P240

**Published:** 2013-07-08

**Authors:** Ching-Ling Teng, William B Levy

**Affiliations:** 1 Departments of Psychology and of Neuroscience, University of Virginia, Charlottesville, VA 22904, USA; 2 Departments of Neurosurgery and of Psychology, University of Virginia, Charlottesville, VA 22908, USA

## 

A simple stimulus evokes responses from a large population of neurons in many cortical areas. However, although many neurons are active, not all contribute equally to perception or motor planning. Studies of motion discrimination show that an animal's perceptual decisions are well correlated with responses from a relatively small fraction of MT neurons. There are similar findings in other systems. Such a subset of neurons is labeled "most informative", and arguably is the basis of a perceptual decision [1,2].

In this study, we use a simple model of two (or two pools of) competing neurons to find the location of the "most informative" neurons in the context of error-minimization for a broad range of discrimination tasks. Although the peak and the maximum slope of a tuning curve are typically emphasized in sensory coding theories, the quantitative interaction described here requires one to consider the entire tuning curve. We start the analysis with a fine discrimination task, but the theory is general enough for a continuum of discrimination tasks, from fine to coarse.

**Figure 1:**
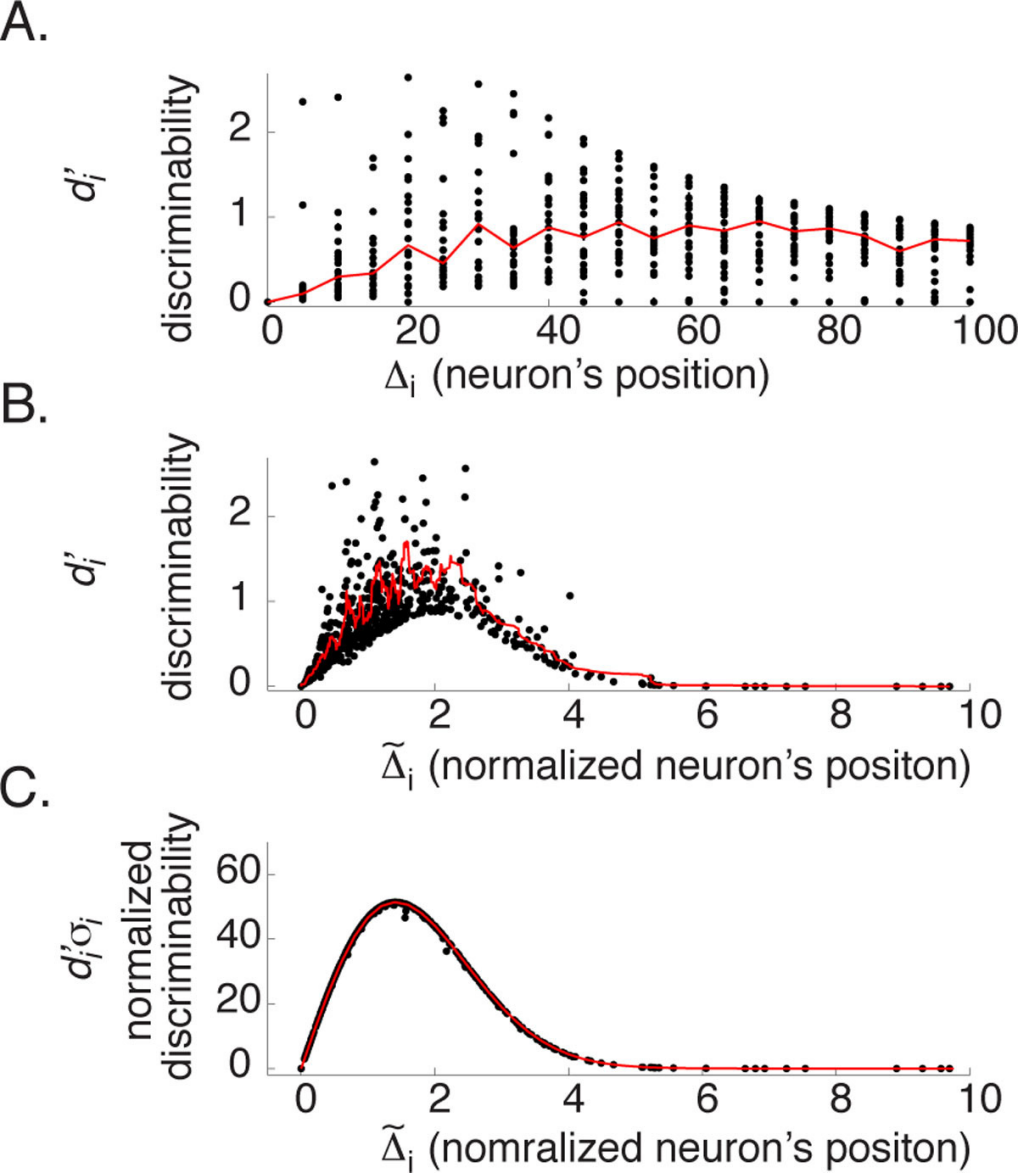
**Reinterpreting experimental observations**. (A) A single neuron's performance (expressed as discriminability, $d_{i}^{\prime }$) to a discrimination task (± 3° motion discrimination) is typically expressed as a function of a neuron's tuned position (expressed as the offset ${\text{Δ}}_{i}$from the stimulus). Although the dataset here is synthetic, it is based on empirical values [2,3]. Notably in both cases, the data are highly variable: the median (red curve) both peaked near 70° but the central tendency is weak. (B) Rescaling a neuron's position by its own tuning width ${\tilde{\text{Δ}}}_{i}:={\text{Δ}}_{i}/\sigma_{i}$ reduces variation in two ways: a stronger central tendency along the x-axis, and substantially reduced variability along the y-axis. The red curve is the running average of 20 successive data points. (C) Variability with this same dataset is further reduced if $d_{i}^{\prime }$ is also scaled as $D^{\prime }:=d_{i}^{\prime }\cdot \sigma_{i}$. All plots are based on the same 620 neurons. Offsets ${\text{Δ}}_{i}$are uniformly sampled from 0 to 100° in 5° increments with 30 repeats. Tuning widths are sampled from a Gaussian distribution (mean = 47°; standard deviation = 27°). A Poisson random number generator produced the spike count.

The results point out that (1) nearly any place on a tuning curve (between the peak and 1.4 tuning widths from the peak) can be an optimal position for a particular discrimination problem; (2) multiplicative noise does not alter the location of the most informative neuron, while additive noise slightly alters the location; (3) the maximum slope of a neuron's tuning curve is generally suboptimal for a fine discrimination task. In fact, the optimal stimulus location for fine discrimination is farther into the tail of a tuning curve under most conditions; (4) even as the optimal stimulus position on a tuning curve is shifting with the task, the most informative neuron remains unchanged over a broad range of discrimination. Finally, we show practically how use the theory to bring order to what appears as discouragingly noisy data (see Figure 1).
